# Evaluation of Therapeutic Oligonucleotides for Familial Amyloid Polyneuropathy in Patient-Derived Hepatocyte-Like Cells

**DOI:** 10.1371/journal.pone.0161455

**Published:** 2016-09-01

**Authors:** Christoph J. Niemietz, Vanessa Sauer, Jacqueline Stella, Lutz Fleischhauer, Gursimran Chandhok, Sarah Guttmann, Yesim Avsar, Shuling Guo, Elizabeth J. Ackermann, Jared Gollob, Brett P. Monia, Andree Zibert, Hartmut H. -J. Schmidt

**Affiliations:** 1 Klinik für Transplantationsmedizin, Universitätsklinikum Münster, Albert-Schweitzer-Campus 1, Gebäude A14, D-48149, Münster, Germany; 2 Ionis Pharmaceuticals, Inc., 2855 Gazelle Ct, Carlsbad, CA, 92010, United States of America; 3 Alnylam Pharmaceuticals, Inc., 300 Third St #3, Cambridge, MA, 02142, United States of America; Rutgers University Newark, UNITED STATES

## Abstract

Familial amyloid polyneuropathy (FAP) is caused by mutations of the transthyretin (*TTR*) gene, predominantly expressed in the liver. Two compounds that knockdown TTR, comprising a small interfering RNA (siRNA; ALN-TTR-02) and an antisense oligonucleotide (ASO; IONIS-TTR_Rx_), are currently being evaluated in clinical trials. Since primary hepatocytes from FAP patients are rarely available for molecular analysis and commercial tissue culture cells or animal models lack the patient-specific genetic background, this study uses primary cells derived from urine of FAP patients. Urine-derived cells were reprogrammed to induced pluripotent stem cells (iPSCs) with high efficiency. Hepatocyte-like cells (HLCs) showing typical hepatic marker expression were obtained from iPSCs of the FAP patients. *TTR* mRNA expression of FAP HLCs almost reached levels measured in human hepatocytes. To assess TTR knockdown, siTTR1 and TTR-ASO were introduced to HLCs. A significant downregulation (>80%) of *TTR* mRNA was induced in the HLCs by both oligonucleotides. TTR protein present in the cell culture supernatant of HLCs was similarly downregulated. Gene expression of other hepatic markers was not affected by the therapeutic oligonucleotides. Our data indicate that urine cells (UCs) after reprogramming and hepatic differentiation represent excellent primary human target cells to assess the efficacy and specificity of novel compounds.

## Introduction

Transthyretin (TTR) related amyloidosis (ATTR) represents a class of life-threatening and progressing diseases that is associated with the misfolding of TTR, a major blood protein and a carrier of retinol (vitamin A) and thyroxine (T4) [1]. TTR is primarily (>95%) produced in the liver as a tetramer. Amyloid aggregation is believed to be a result of decreased tetramer stability resulting in dissociation of TTR into monomers [2,3]. *In vitro* studies revealed that monomeric TTR is prone to unfolding and ultimately followed by self-assembly into oligomers and amyloid fibrils [4,5]. Amyloid TTR fibrils are frequently found in peripheral neurons, gastrointestinal tract and heart. In hereditary ATTR with polyneuropathy, also known as familial amyloid polyneuropathy (FAP), the peripheral nerves are primarily affected, while in cardiomyopathy-related TTR amyloidosis, also known as familial amyloid cardiomyopathy (FAC), neuropathy is usually less prominent or even absent. Patients mostly develop a severe disease and die within 5 to 15 years after onset. While these ATTR forms can be ascribed to a dominant expression of the *TTR* gene variants, only wild type TTR is expressed in senile systemic amyloidosis (SSA), a type of amyloidosis frequently found in elderly people [6,7].

Until recently, the only treatment option for patients having FAP was liver transplantation. Of note, transplantation results in the inhibition of variant TTR synthesis, while the wild type TTR is produced at a high level [8]. Unfortunately, there is a limited availability of organs and transplantation is associated with significant morbidity. At an early stage of the disease, FAP patients carrying the ATTRV30M variant benefit the most from transplantation. However, worsening of the disease, e.g. neuropathy, is frequently observed in the recipients over time [9]. Moreover, amyloid deposition continues in the patients indicating that variant TTR is no longer responsible for progression of the disease. An alternate therapy includes small molecules, such as Tafamidis. Tafamidis has been approved in Europe for the therapy of adult FAP patients with stage 1 polyneuropathy, targeting the stabilization of the TTR tetramer. The progression of the disease was shown to be reduced after administration of Tafamidis [10–12].

Suppression of the variant TTR synthesis by interference with mRNA has been reported for ribozymes demonstrating the feasibility of this approach [13,14]. However, biochemical and clinical evidence suggests that the wild type TTR can also significantly contribute to the disease [15,16]. It is therefore conceivable that mitigating the overall TTR synthesis can address the current demand for an efficient therapy of ATTR. Small interfering RNAs (siRNAs) and antisense oligonucleotides (ASOs) are the most commonly used strategies for silencing gene expression [17,18]. ASOs are short single-stranded stretches of DNA or RNA with complementary sequence to their target mRNA, while siRNAs are double-stranded and afford activation by the enzyme complex Dicer. Due to the advances in the modification of the oligonucleotides, including changes to the nucleotide chemistry that increase the resistance of the oligonucleotides to degradation, siRNAs and ASOs have recently evaluated in clinical trials [19–22]. For therapy of FAP, two novel compounds, ALN-TTR02 and IONIS-TTR_Rx,_ are currently under clinical investigation [23–28]. ALN-TTR02 is a lipid nanoparticle-formulated siRNA [29], whereas IONIS-TTR_Rx_ is a second generation antisense gapmer, both targeting human variant and wildtype *TTR* mRNA.

Induced pluripotent stem cells (iPSCs), mostly derived from fibroblasts, have been postulated to model disease [30] and were also used to generate hepatocyte-like cells (HLCs) of FAP patients [31,32]. Recently, urine cells (UCs) were reported as a novel source for reprogramming [33–36]. Isolation of UCs for generation of iPSCs exhibits several advantages (i) accessibility is given at any time point as urine is an inexhaustible source, (ii) procedures are independent from age and gender, (iii) cell isolation techniques are simple and UCs can be easily expanded using standard cell culture conditions, (iv) procedures are excellently suited to be integrated into the routine clinical practice. We have therefore utilized UCs as a patient-friendly source for somatic cell isolation. In this study, we addressed whether FAP patient-derived UCs are suited to obtain a set of HLCs, representing various geno- and phenotypes of the disease, for the evaluation of novel TTR-specific siRNA and ASO compounds.

## Materials and Methods

### Ethics Statement

All experiments were approved by the ethics committee of the University Clinic of Muenster and written informed consent was obtained from all patients and individuals enclosed in the study. The characterization of FAP patients is given according to the previously suggested nomenclature [37].

### Isolation and Cultivation of UCs

Spontaneously voided urine was collected and processed as described [38,39]. One well of a 12-well cell culture plate (Greiner Bio-One) was precoated with 0.1% gelatin (Millipore). Urine samples were centrifuged at 400 x g for 10 minutes. Cell pellets were washed with PBS (Sigma-Aldrich) / 1% penicilline-streptomycine (PAA) and resuspended in 1 ml of UC medium consisting of DMEM/F-12 (Gibco), 10% FCS (PAA), 1% penicilline-streptomycine (PAA), MEM non-essential amino acid solution (NEAA, Sigma-Aldrich), 1x GlutaMAX-I (Gibco), 0.1 mM 2-Mercaptoethanol (Gibco) and SingleQuot Kit CC-4127 REGM (Lonza). Cells were incubated at 37°C, 5% CO_2_ for 24 hours. 1 ml of fresh UC medium was added for three days. Half of the media was changed every second day. At a cell confluence of 80–90%, cells were washed and detached using trypsin/EDTA (Gibco). After addition of trypsin inhibitor (DTI, Gibco), cells were resuspended in 2 ml of UC medium.

### Reprogramming of UCs

At a cell confluence of 80–90%, UCs were harvested by trypsinization. 0.5 x 10^6^ cells were reprogrammed using the Amaxa Basic Nucleofector Kit for Primary Mammalian Epithelial Cells (Lonza, VPI-1005) according to the protocol of the manufacturer. 1 μg of each vector pCXLE-hOCT3/4-shp53-F, pCXLE-SK and pCXLE-hUL (addgene) was used. The cells were transferred to the Matrigel^®^ matrix (Corning) coated well. After 24 hours, the UC medium was replaced by mTeSR-1 (Stemcell Technologies), supplemented with 1% penicilline-streptomycine and changed every second day. Cells were subcultured every 5 to 7 days. For further passaging, iPSCs were dissociated with 1 U/ml Dispase (Stemcell Technologies).

### DNA Sequencing

For detection of patient-specific *TTR* variant, the chromosomal DNA was isolated using QIAamp DNA mini kit (Qiagen) and the genotype was determined by DNA sequencing using Big Dye Version 3.1 (Life Technologies).

### Immunocytochemistry

Cells were fixed with 4% paraformaldehyde (PFA, Electron Microscopy Sciences) for 30 minutes, washed with PBS and permeabilized with 0.5% Triton X-100 (Fisher Scientific) in PBS for 15 minutes. Cells were blocked with 3% BSA (Sigma-Aldrich), followed by incubation at 4°C with primary antibody (S1 Table). After washing, cells were incubated with a secondary antibody for 1 hour. Cells were counterstained with DAPI (Sigma-Aldrich) and visualized (Olympus CKX41-X10; cellSens Standard 1.11 imaging software).

### Real-Time qRT-PCR

Total RNA was isolated using RNeasy kit (Qiagen). For first-strand cDNA synthesis, 1 μg of RNA was applied to SuperScript II Reverse Transcriptase (Invitrogen) according to the instructions of the manufacturer. 1 μl of the reverse transcriptase product was mixed with SYBER Green PCR Core Plus (Eurogentec) and 150 nM of primers (S2 Table) were added. PCR was analyzed on the ABI Prism 7900 HT Sequence Detection System (PE Applied Biosystems) by using a 3-stage program for 2 min at 50°C, 10 min at 95°C, and 40 cycles of 14 sec at 95°C, 1 min at 60°C. Data were analyzed by SDS 2.4 software (PE Applied Biosystems). Ct values were normalized to *GAPDH* or *UBQLN1* (2^-ΔΔCt^). As an internal control, RNA was derived from primary human hepatocytes (PHH) derived from donor organs after whole liver perfusion and cryopreservation.

### Embryoid Body Formation

**i**PSCs were treated with Dispase to generate small iPSC clusters. Cell clusters were centrifuged at 200 x g for 5 minutes, resuspended in 2 ml embryoid body medium as described in the manual (TaqMan^®^ hPSC Scorecard^™^) and transferred to one well of 6-well suspension culture plate (Greiner Bio-One). The medium was changed daily and after 7 days of cultivation, cell pellets were collected for RNA isolation. Evaluation of the trilineage differentiation potential of iPSCs was examined using the 384-well hPSC Scorecard^™^ Panel (Life Technologies) and evaluated by the Thermo Fisher Cloud hPSC Scorecard Analysis software.

### Generation of HLCs

Hepatic induction of iPSCs was accomplished as described previously with minor modifications [39–41]. In brief, single cells were generated after Accutase incubation (Sigma-Aldrich) for 6–8 minutes. Cells were resuspended in mTeSR-1 and transferred to Matrigel-precoated cell culture plates. After 24 hours, culture medium was changed for the next 3 days to DMEM/F12 supplemented with 100 ng/ml recombinant Activin-A (R&D Systems), 100 ng/ml fibroblast growth factor-2 (FGF2, Peprotech), 50 ng/ml recombinant human Wnt3a (R&D Systems). Concentration of KnockOut SR Xenofree CTS (KSR, Gibco) was 0%, 0.2% and 2.0% for the first, second and third day, respectively. For the next 8 days, cells were cultivated in DMEM/F12 supplemented with 10% KSR, 1mM NEAA, 1mM L-Glutamine, 1% dimethyl sulfoxide (DMSO, Sigma-Aldrich), and 100 ng/ml hepatocyte growth factor (HGF, Peprotech). Finally, cells were grown for 3 days in 10% KSR, 1mmol/L NEAA, 1mM L-Glutamine, and 0.1μM dexamethasone (Sigma-Aldrich).

### Flow Cytometry

1 x 10^6^ cells were fixed with 4% PFA for 30 minutes, washed with PBS and permeabilized. After blocking, a 1:100 dilution of antibody was added for 30 minutes. Secondary antibody staining (1:100) was performed for 30 minutes. Cells were analyzed in a Coulter Epics XL-MCL (Beckman Coulter).

### Periodic Acid-Schiff Staining

At day 14 of hepatic differentiation, cells were analyzed for glycogen synthesis and storage by Periodic Acid-Schiff (PAS) staining using the system protocol (Sigma-Aldrich, 395B-1KT).

### Analysis of Cell Culture Supernatants

Total supernatant was separated on a 12.5% SDS polyacrylamide gel, and blotted onto Amersham Protran Premium 0.45 NC (GE Healthcare Life Sciences). The membrane was blocked overnight at 4°C in Tris-buffered saline (TBS) containing 0.05% Tween 20 (TBS-T, Sigma-Aldrich) and 5% non-fat dry milk (AppliChem). After washing with 0.05% TBS-T, the membrane was incubated with a polyclonal rabbit anti-TTR antibody (S2 Table) diluted 1:500 and incubated for two hours at room temperature. Horseradish peroxidase (HRP)-linked secondary antibody was used (GE Healthcare Life Sciences). Amersham ECL Western Blotting Detection Reagent (GE Healthcare Life Sciences) was added. Blots were quantified using ImageJ 1.48v analysis software. TTR of cell culture supernatants was analyzed using liquid chromatography-high resolution accurate mass MS (LC-HRAM MS) in positive-ion mode.

### siRNA and ASO Incubation

siTTR1 and TTR-ASO were generated according to the procedures used for synthesis of ALN-TTR02 and IONIS-TTR_Rx_, respectively. At day 14 of hepatic differentiation, the medium of HLCs was changed to Opti-MEM (Gibco). Lipid nanoparticle-formulated siTTR1 was added at 0.3 μM to the medium. TTR-ASO (1.5 μM) was complexed with 2.5 μl Lipofectamine^®^ 2000 Reagent (Life Technologies) prior to transfer to the culture medium of HLCs. The culture medium was changed daily.

### Statistical Analysis

Statistical analysis was performed by Kruskal-Wallis 1-way ANOVA and Student’s t-test using SPSS 22.0 software. Data are given as mean ± standard error (SE).

## Results

### Isolation of Urine Cells from FAP Patients

**Fig 1:**
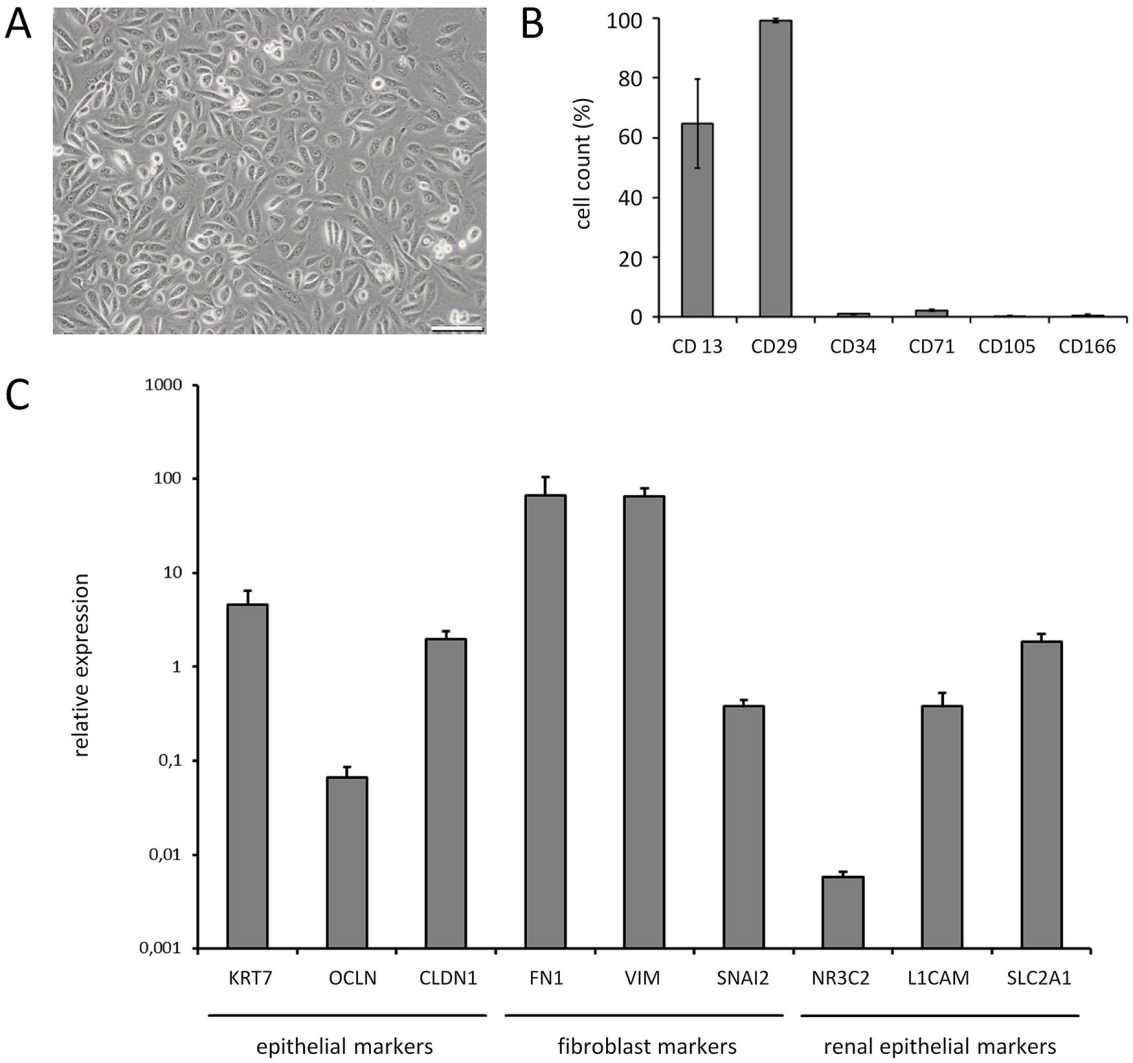
Characterization of urine cells. (A) Brightfield image of UCs at p2 after isolation. FAP3 derived cells are shown as one typical example. Scale bar, 100 μM. (B) Flow cytometry analysis of UCs at p2 (n = 4). Total expression of markers used for characterization of mesenchymal stem cells is given. (C) Relative mRNA expression of epithelial, fibroblast and renal marker genes in UCs (n = 6). Data were normalized to housekeeping gene.

Urine samples of 21 FAP patients (8 female, 13 male, mean age 52.1 ± 13 years) were processed for isolation and cultivation of renal tubular cells. In total, outgrowth of UCs was achieved with 12 FAP patients (57.1%). The efficacy of UC cultivation could be increased when a second donation was available. Urine from healthy donors (n = 10; mean age 43.0 ± 16) showed a somewhat higher efficacy (70.0%) [38,42,43]. Female and male FAP donors showed almost the same efficacy of UC isolation (50.0% and 61.5%, respectively). The age of FAP patients, the *TTR* variant or the absolute volume of the urine donation did not affect the efficacy of UC generation (data not shown). Of note, 80 ml of urine was sufficient for generation of UCs in one FAP patient. Typically, 2 to 6 million UCs were obtained after 2 to 3 weeks at a passage number of 2 (p2). Contamination of the UC-derived cell cultures were observed in two preparations (6.9%). The morphology of the UCs presented a typical smooth-edged shape and cobble stone-like appearance (Fig 1A) and immunocytochemistry stainings indicated the expression of epithelial and renal proximal tubular markers (data not shown) confirming similar observations in previous reports [33]. UCs were recently reported to express markers of mesenchymal stem cells [42]. A flow cytometry analysis of the UCs was performed using markers typically present or absent on mesenchymal stem cells [44], e.g. CD13, CD29, CD71, CD105, CD166 (present) and CD34 (absent). Epithelial markers CD13 and CD29 were found to be highly expressed in UCs whereas CD34, CD71, CD105 and CD166 were not detected (Fig 1B). qRT-PCR analysis confirmed a high expression of epithelial, fibroblast and renal markers in the UCs (Fig 1C).

### Reprogramming of UCs from FAP Patients

**Fig 2:**
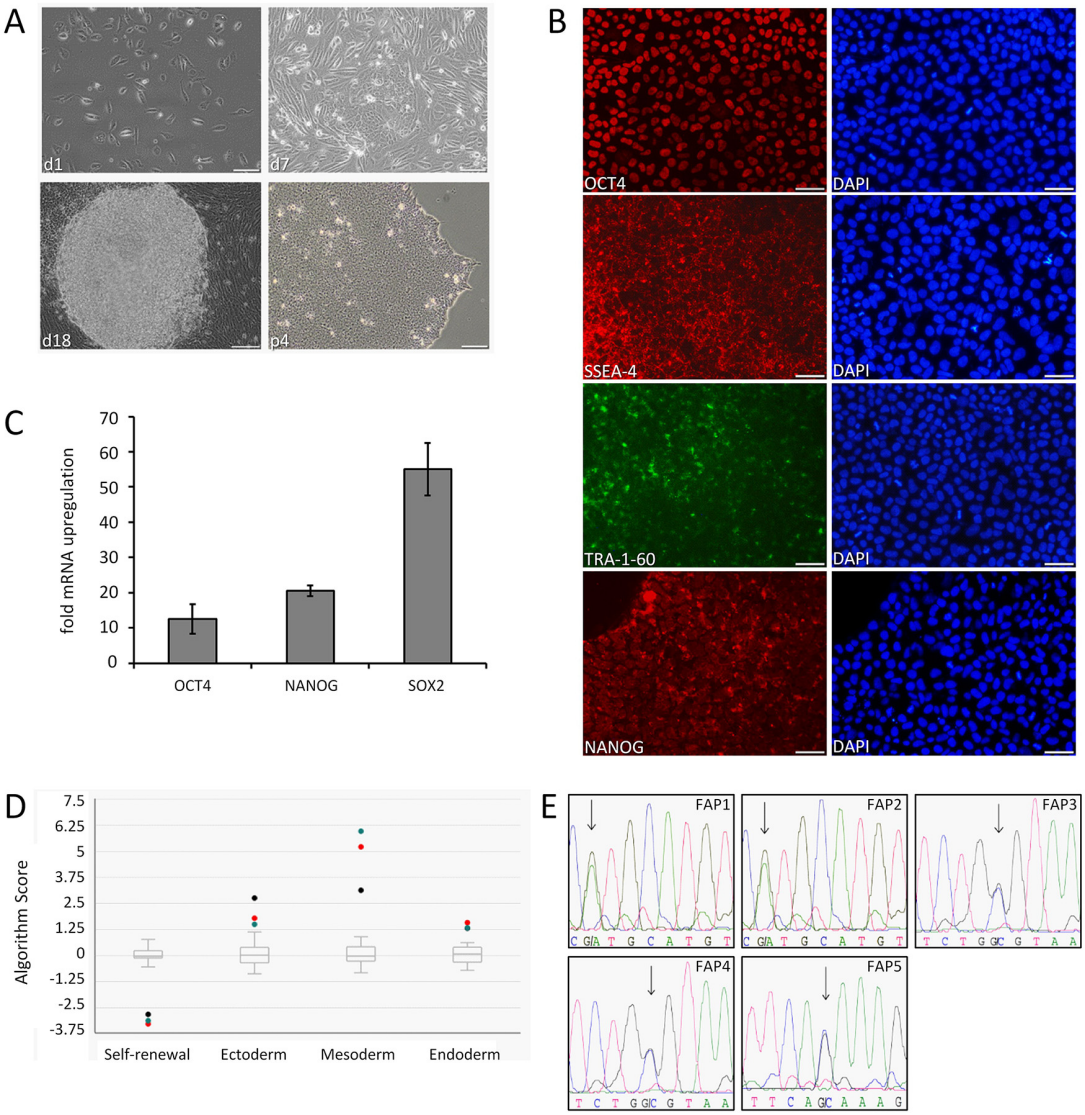
Reprogramming of urine cells obtained from FAP patients. (A) Typical brightfield images of UCs from a FAP patient at different times after reprogramming. FAP2 derived cells are shown. Scale bars, 100 μM. (B) Immunofluorescence stainings of cells after reprogramming. DAPI was used for nuclear counterstaining. FAP5 derived cells are shown as one typical example. Scale bars, 50 μM. (C) Gene expression analysis of reprogrammed cells. Relative expression to housekeeping gene is shown. Mean±SE of FAP1 to FAP5 are shown. (D) EB expression profile of pluripotency- and lineage-specific markers derived from FAP iPSCs. Dots indicate sample scores of cells derived from patients FAP1 (black), FAP2 (red) and FAP4 (green). Human embryonic stem (hES) and iPS cell lines served as control (box plot). (E) DNA sequencing chromatograms of the *TTR* gene derived from FAP iPSCs. Heterozygotic variant nucleotides are shown by arrow.

UCs from five FAP patients at p2 were selected for reprogramming (Table 1). Two of the five FAP patients express ATTRV30M which represents the most frequent *TTR* variant worldwide. The variants ATTRG47A and ATTRR34T are found worldwide and have a high prevalence in German FAP patients. The selected FAP patients show various clinical manifestations, either presymptomatic or with early and late onset of disease. The UCs were reprogrammed by transient expression of the factors OCT4, SOX2, KLF4, L-MYC, LIN28, and an shRNA against p53 [39,45]. The resulting cell lines gave rise to typical iPSC-like colonies within 10 to 20 days after nucleofection as judged by morphology (Fig 2A). After reprogramming, the pluripotent character of the cells was assessed by immunocytochemistry for the expression of embryonic stem (ESC) cell-specific markers OCT3/4, SSEA-4, TRA-1-60 and NANOG (Fig 2B). Real-time qRT-PCR analysis revealed that OCT3/4, NANOG and SOX2 were highly expressed in the cells (Fig 2C). To further assess the pluripotency of the reprogrammed cells, formation of embryoid bodies (EB) was employed. Trilineage differentiation potential of the urine-derived FAP cells was close to a set of 13 functionally characterized hES and iPS reference cell lines [46] with a modest bias to the mesodermal lineage (Fig 2D; S1 Fig). To confirm that the *TTR* variant was not altered in the reprogrammed cells, the chromosomal DNA was isolated and subjected to sequence analysis (Fig 2E). The 3’ end of the *TTR* gene which is indispensable as a target for knockdown was also analyzed by DNA sequencing (data not shown). In summary, the data reveal that reprogramming of the UCs obtained from FAP patients result in high expression of pluripotency markers, typical of iPSCs, and retention of *TTR* variant gene sequences. Two clones of iPSCs were examined throughout the study.

**Table 1:** Characteristics of FAP patients.

Name	Gender	Age	Variant	Phenotype
FAP1	F	33	ATTRV30M	Neuropathy, early onset
FAP2	F	70	ATTRV30M	Neuropathy, late onset
FAP3	F	54	ATTRG47A	Presymptomatic
FAP4	M	51	ATTRG47A	Cardiac, neuropathy
FAP5	M	59	ATTRR34T	Neuropathy, cardiac

### Differentiation of FAP iPS Cells toward Hepatocyte-Like Cells

**Fig 3:**
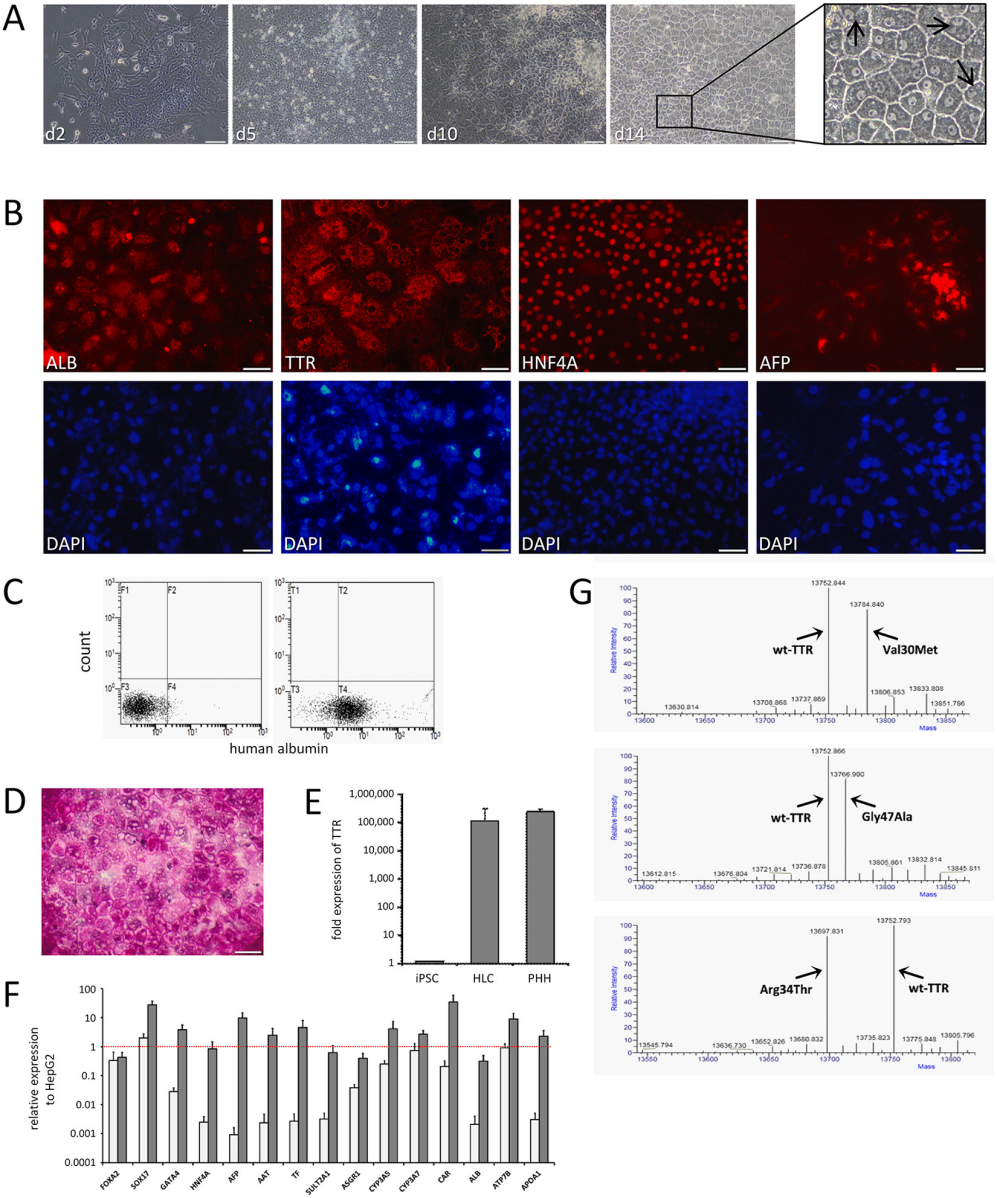
Generation of FAP iPS cell-derived hepatocyte-like cells. (A) Brightfield images of iPSCs at different days after *in vitro* differentiation. FAP5 derived cells are shown as one typical example. Scale bars, 100 μM. Arrows in the zoomed area point to binucleated cells. (B) Immunofluorescence stainings of cells at day 14 of differentiation. FAP4 derived cells are shown as one typical example. Scale bars, 50 μM. (C) Albumin flow cytometry analysis cells at day 14. FAP1 derived cells are shown as one typical example. (D) PAS staining of HLCs to indicate glycogen synthesis. FAP4 derived cells are shown as one typical example. (E) *TTR* mRNA expression in HLCs. Mean±SE of FAP1 to FAP5 are shown. Primary human hepatocytes (PHH) are shown as control. Mean expression was normalized to housekeeping gene. (F) qRT-PCR analysis of FAP HLCs (grey) and respective iPSCs (white). Mean±SE of FAP1 to FAP5 are shown. Dotted line indicates same expression relative to human hepatoma HepG2 reference cells line. (G) Mass spectral analysis of cell culture supernatants of HLCs derived from patients FAP1, FAP3 and FAP5 are shown. Arrows indicate peaks for TTR wild type and variant form.

The five generated FAP iPS cell lines were subjected to a hepatic differentiation protocol as described previously [39,40]. The cell morphology changed significantly within the first two days of treatment (Fig 3A). Gene expression analysis indicated a major change of the expression levels for pluripotency and hepatic markers (S2 Fig). At day 14 of hepatic differentiation, cells displayed a polygonal shape, binucleation, and an increased nuclear-cytoplasmic ratio typical for human hepatocytes (magnification given in Fig 3A). Immunocytochemistry stainings for human albumin, TTR, HNF4a and AFP indicate that the expression of major hepatocyte-specific markers was upregulated in the cells (Fig 3B). Flow cytometry analysis (Fig 3C) confirmed human albumin expression in up to 90.1% of the cells (mean 70.7 ± 18). The glycogen synthesis of the differentiated cells was assessed by PAS staining, indicating the presence of a major function of human hepatocytes (Fig 3D). Notably, *TTR* mRNA expression was highly expressed in differentiated cells at day 14 (Fig 3E). *TTR* upregulation was several orders of magnitude higher as compared to iPSCs and almost reached the expression level of human hepatocytes. qRT-PCR analysis revealed the upregulation of definitive endodermal and various hepatocyte marker genes as compared to iPSCs (Fig 3F). Mass spectral analyses of cell culture supernatants derived from the differentiated cells identified both, the secreted wild-type TTR and the mutant TTR form at almost equal ratios (Fig 3G). The data indicate that differentiation of iPSCs derived from UCs of FAP patients result in hepatocyte-like cells (HLCs) that are excellently suited to study the effect of therapeutic oligonucleotides directed against TTR.

### TTR Knockdown in FAP HLCs by siRNA and Antisense Clinical Study Compounds

**Fig 4:**
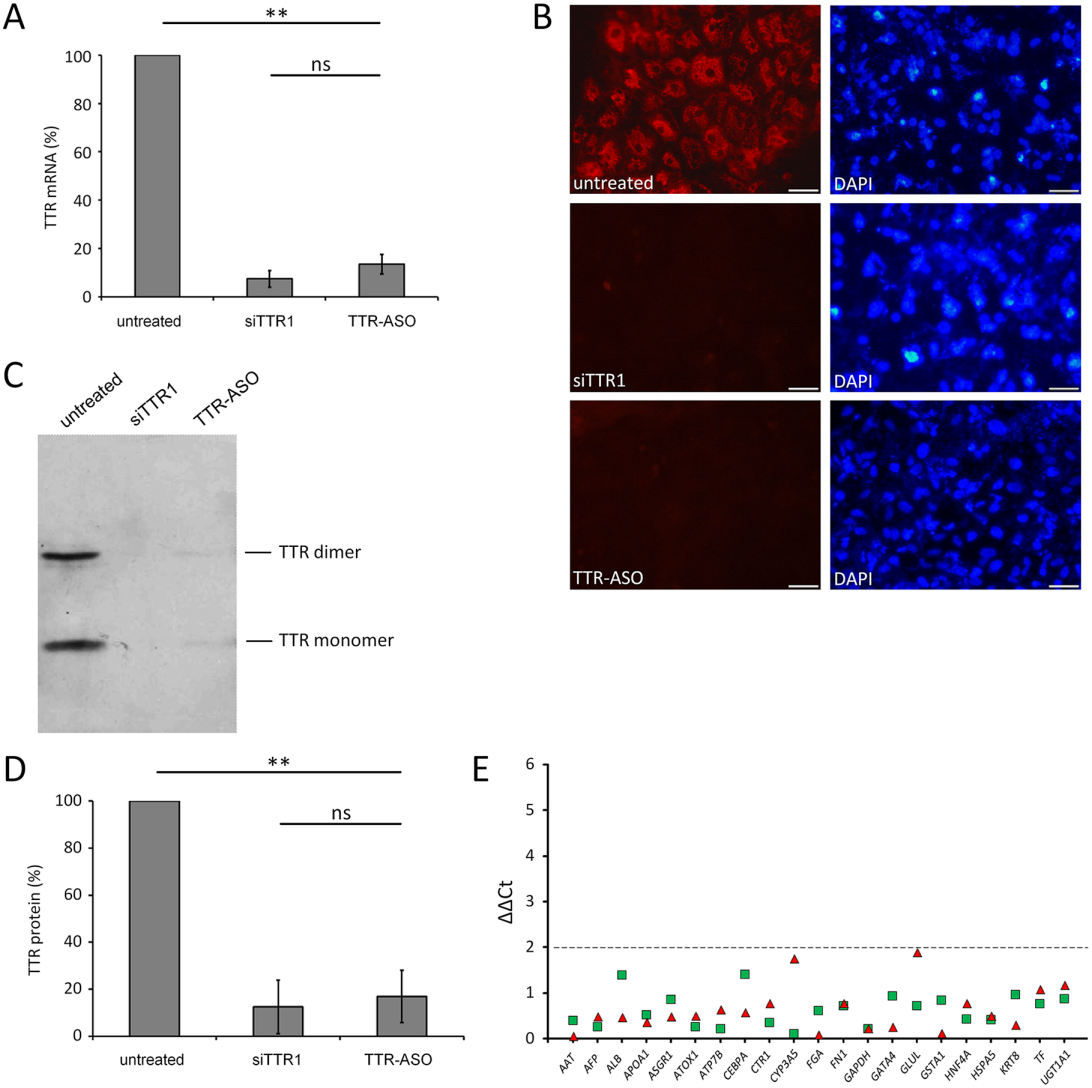
TTR knockdown in FAP patient derived hepatocyte-like cells. (A) qRT-PCR analysis of *TTR* mRNA expression in FAP HLCs treated with compounds (n = 5). Mean±SE of FAP1 to FAP5 are shown. (B) Immunocytochemistry stainings of TTR in FAP HLCs 24 hours after treatment with compounds. FAP4 derived cells are shown as one typical example. Exposure time was consistently adjusted to 1/6 second. Scale bars, 50 μM. (C) TTR Western blot analysis of cell culture supernatants derived from HLCs. FAP4 derived cells are shown as one typical example. (D) Quantification of TTR expression as obtained by Western blots. Mean±SE of FAP1, FAP4 and FAP5 are shown. (E) qPCR analysis of different hepatic and housekeeping marker genes in FAP HLCs after treatment with siTTR1 (triangle) and TTR-ASO (squares). Mean of FAP1 to FAP5 are shown. Data were normalized and set relative to untreated control cells. Absolute ΔΔCt-values are given. Dotted line indicates significance level. ** p< 0.01; ns, not significant.

The siRNA and antisense compounds ALN-TTR02 and IONIS-TTR_Rx_ that are directed against human *TTR* are currently being evaluated in clinical studies for therapy of FAP patients [23,26]. The efficacy of these compounds was assessed in the established five HLCs. An almost complete silencing of *TTR* mRNA was observed in all five HLCs after 24 hours. Knockdown of *TTR* mRNA by siTTR1 and TTR-ASO was about 80 to 90% (Fig 4A) whereas scrambled control oligonucleotides did not show any significant downregulation (data not shown). Comparison of *TTR* knockdown between individual FAP cell lines revealed minor variability (S3 Fig). Both compounds were equally effective in HLCs derived from healthy individuals suggesting that wildtype *TTR* mRNA is targeted with the same efficacy (S4 Fig). Assessment of cell-associated TTR expression by immunocytochemistry suggested that both compounds almost completely blocked TTR protein synthesis, while at the same time albumin and alpha-fetoprotein expression remained unchanged(Fig 4B; S5 Fig). To validate the impact of the compounds on secreted TTR, a Western blot analysis was performed using the cell culture supernatants derived from the HLCs. Both the monomeric and the dimeric form of TTR were significantly downregulated after addition of the two compounds (Fig 4C). Densitometric quantification of TTR-specific bands revealed a downregulation between 80% to 90% (Fig 4D). The non-specific off-target effects of siTTR1 and TTR-ASO were determined in FAP HLCs by qRT-PCR analysis. Various liver-specific genes encoding proteins of human hepatocytes, including albumin, transferrin and fibronectin, and housekeeping genes (glyceraldehyde 3-phosphate dehydrogenase, keratin 8 and copper transport 1) were assessed. None of the genes showed a significant up- or downregulation in the FAP HLCs after the addition of both compounds indicating a high specificity and a low off-target effect (Fig 4E).

## Discussion

Due to invasive procedures, primary cells, e.g. hepatocytes, are rarely available for many applications without ethical concerns, including the evaluation of novel compounds that have a high potential to enter clinical application. In this study, the molecular evaluation of novel compounds for the therapy of FAP patients was assessed in a stem cell-based model. In recent reports, UCs have been isolated from healthy individuals and patients with a high efficiency of up to 82% [38,42,43,47]. The efficacy of somatic cell isolation from the urine observed in our cohort of 21 FAP patients seemed to be moderately lower indicating that disease-specific determinants may modulate the isolation process. However, as cell isolation could be easily repeated from consecutive donations, a high overall efficacy can be achieved from urine [38]. In addition, contamination of only two UC cultures has been observed in the study. Contamination could be further reduced when the cell culture media was supplemented with antibiotics (data not shown) suggesting that bacterial contamination does not significantly impede efficacy of the procedure. The marker gene expression profile of the cultivated UCs seems to be diverse, since uroepithelial progenitor, pericyte, renal and mesenchymal markers are upregulated in the UCs [33,42,48]. Our characterization of the UCs derived from FAP patients showed high epithelial, fibroblast and renal marker gene expression. Differences of gene expression profiles may depend on the protocols that were used for UC isolation and propagation or based on the individual patient cohort. While different cell types derived from urine have been observed earlier [38,42], the likely heterogenous origin of the UC population, e.g. epithelial cells of urinary bladder, squamous epithelial cells and ureter epithelial cells amongst others,as well as the consequences for reprogramming remains to be further explored.

We routinely observed first iPSC-like colony formation around day 10 after reprogramming of the FAP patient derived UCs using plasmids that encode EBV elements as also reported by others [49,50]. The similarity of iPSCs and UCs with regard to their epithelial state might therefore accelerate reprogramming as compared to fibroblasts that typically form iPSC-like colonies around week 3 to week 4 [49]. It has been argued that epithelial cells, e.g. keratinocytes, do not have to undergo mesenchymal-to-epithelial transition (MET), a possible intermediate of the reprogramming events [51–54]. Of note, after reprogramming of the FAP UCs, a relative high expression of mesodermal markers was observed when iPSCs were regrown in suspension culture to form EBs. Whether this represents an epigenetic conservation of the UCs or is due to the reprogramming factors has to be further investigated.

The protocol of hepatic differentiation used in our study resulted in HLCs that share many characteristics of hepatocytes. However, the characterization of HLCs in our and other studies also revealed the expression of premature hepatocyte marker, e.g. *AFP*, suggesting that HLCs generated from iPSCs represent immature fetal hepatocytes [31,32,35,39,55]. With regard to *TTR* expression, our results suggest that at day 3 of HLC differentiation, where endodermal markers were highly expressed, *TTR* was not induced. However, it cannot be excluded that *TTR* expression of HLCs at d14 may partly originate from premature cells.

One intriguing finding of our study was the high level of *TTR* mRNA expression which was found in the HLCs after hepatic differentiation. The levels almost resembled those of human hepatocytes suggesting that UC derived HLCs represent an ideal cellular model to study the molecular determinants of FAP. In line, both forms of TTR, the wild type and the variant protein, were secreted to almost the same amounts into the cell culture medium, a result that differs from previous findings reported for HLCs that were derived from fibroblasts [32].

The FAP cell culture model established in this study used iPS cell lines from five patients encoding three major TTR variants. Our data demonstrate that siTTR1 and TTR-ASO, were equally effective in reducing TTR expression in the five patient cell lines. While minor differences could be observed between the five FAP cell lines, levels of *TTR* knockdown were significantly reduced in all five patient-derived HLCs. Part of the observed variability could be due to subtle differences of the cell number at the day of treatment. Off-target effects were not observed when analyzing a set of 21 hepatocyte-specific genes. Both compounds target regions located in the highly conserved 3’ UTR of the human *TTR* mRNA, thus interfere with both the wild type and more than 130 *TTR* variants. Although *TTR* variant-specific inhibition by oligonucleotides has been achieved *in vitro* [13,14,56], a downregulation of both TTR forms seems to be favorable from the clinical point of view, since reduction of the overall disease burden in FAP patients seems to involve wild type TTR [8,15,16].

ALN-TTR02 and IONIS-TTR_Rx_ are currently being studied in advanced clinical trials. A highly efficient, long-term downregulation of serum TTR of about > 80% was reported. Concerning the underlying mechanism, both compounds are remarkably different, since ALN-TTR02 is a lipid nanoparticle-formulated siRNA that mediates RNA interference via binding to mRNA/RISC complexes in the cytoplasm, whereas IONIS-TTR_Rx_ represents a naked ASO that is predominantly targeting pre-mRNA in the nucleus via RNaseH [28]. The FAP patients receive both compounds via different application routes. ALN-TTR02 is given by intravenous infusion and IONIS-TTR_Rx_ by subcutaneously injection. Interestingly, at the level of the hepatocyte, the molecular events following administration of the oligonucleotides seem to result in similar efficacy for the TTR knockdown. Of note, although we used similar concentrations of the compounds, our iPSC-based cellular model is limited to address the exact stoichiometry needed for the knockdown, since the naked ASO was delivered by a transfection reagent, whereas the lipid nanoparticle-formulated siRNA could per se mediate cell entry.

To our knowledge this is the first report of an iPSC-based cell model that evaluated compounds currently under clinical investigation. The report suggests that iPSC-based models could be a valuable tool for the validation of novel compounds before they enter the phase of clinical studies.

## Supporting Information

S1 Fig Individual gene expression of FAP iPSCs after embryoid body formation. Relative expression levels of self-renewal, mesendodermal, ectodermal, mesodermal, and endodermal markers in EBs derived from iPSCs of patients FAP1, FAP2 and FAP4 are shown. Lineage-specific mRNA expression was analysed by TaqMan^®^ hPSC Scorecard^™^ Assay. Colors correlate to the fold change relative to a reference set of well characterized ES and iPS cell lines.
(DOCX)

S2 Fig qRT-PCR analysis of FAP HLCs at day 3 of hepatic *in vitro* differentiation. Data from human hepatoma HepG2 cell line (white) are shown as control. Dotted line indicates iPSC gene expression that was used as reference (ΔΔCt). Data were normalized to *GAPDH*. Mean±SE of FAP2 to FAP5 are shown (grey).
(DOCX)

S3 Fig Clonal analysis of *TTR* knockdown in different FAP cell lines. *TTR* knockdown was assessed after treatment (24 h) with siTTR1 (grey) and TTR-ASO (white). HLCs from two iPS cell clones per FAP cell line were analyzed. Data were normalized to *GAPDH*. Untreated cells were set to 100%. Standard deviations are given.
(DOCX)

S4 Fig qRT-PCR analysis of *TTR* mRNA knockdown in HLCs of healthy individuals. Untreated cells were set to 100% (n = 3).
(DOCX)

S5 Fig Protein expression of marker genes following *TTR* knockdown. Immunocytochemistry stainings of albumin and alpha-fetoprotein after treatment with compounds (24 h). One typical experiment derived from HLCs of FAP4 cell line is shown. Exposure time was adjusted to 1/3 second. Scale bars, 50 μM.
(DOCX)

S1 Table Antibodies used in the study. (DOCX)

S2 Table Primer used in the study. (DOCX)
