# Supplementary figures and images for: Evaluation of Therapeutic Oligonucleotides for Familial Amyloid Polyneuropathy in Patient-Derived Hepatocyte-Like Cells
Source: PLoS One. 2016 Sep 1;11(9):e0161455. doi: 10.1371/journal.pone.0161455 (PMC5008816; doi:10.1371/journal.pone.0161455)

**
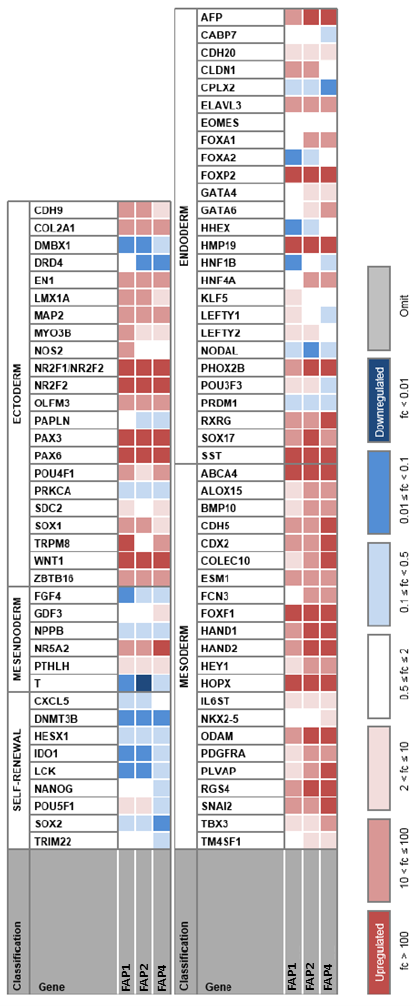
**

**S1 Fig. Individual gene expression of FAP iPSCs after embryoid body formation.**

Supplement: S1 Fig — Relative expression levels of self-renewal, mesendodermal, ectodermal, mesodermal, and endodermal markers in EBs derived from iPSCs of patients FAP1, FAP2 and FAP4 are shown. Lineage-specific mRNA expression was analysed by TaqMan® hPSC Scorecard™ Assay. Colors correlate to the fold change relative to a reference set of well characterized ES and iPS cell lines. (DOCX) [file pone.0161455.s001.docx]

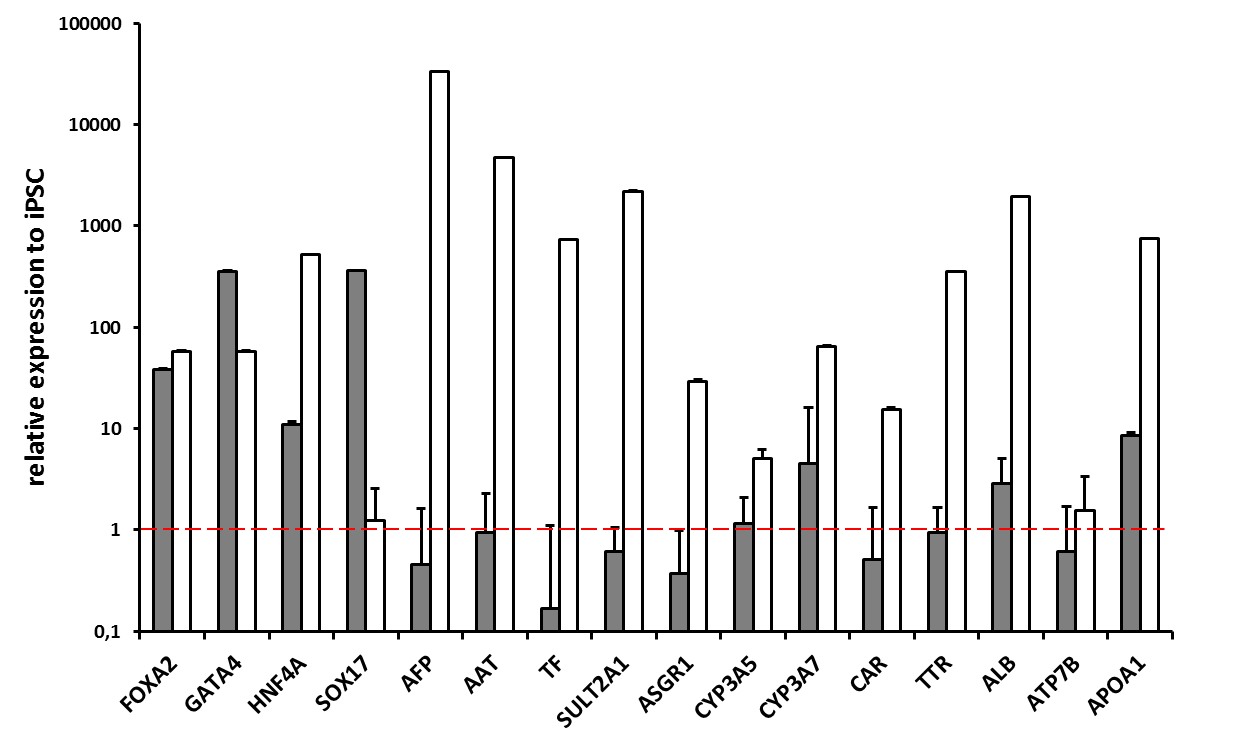
**S2 Fig. qRT-PCR analysis of FAP HLCs at day 3 of hepatic *in vitro* differentiation.**

Supplement: S2 Fig — Data from human hepatoma HepG2 cell line (white) are shown as control. Dotted line indicates iPSC gene expression that was used as reference (ΔΔCt). Data were normalized to GAPDH. Mean±SE of FAP2 to FAP5 are shown (grey). (DOCX) [file pone.0161455.s002.docx]

**
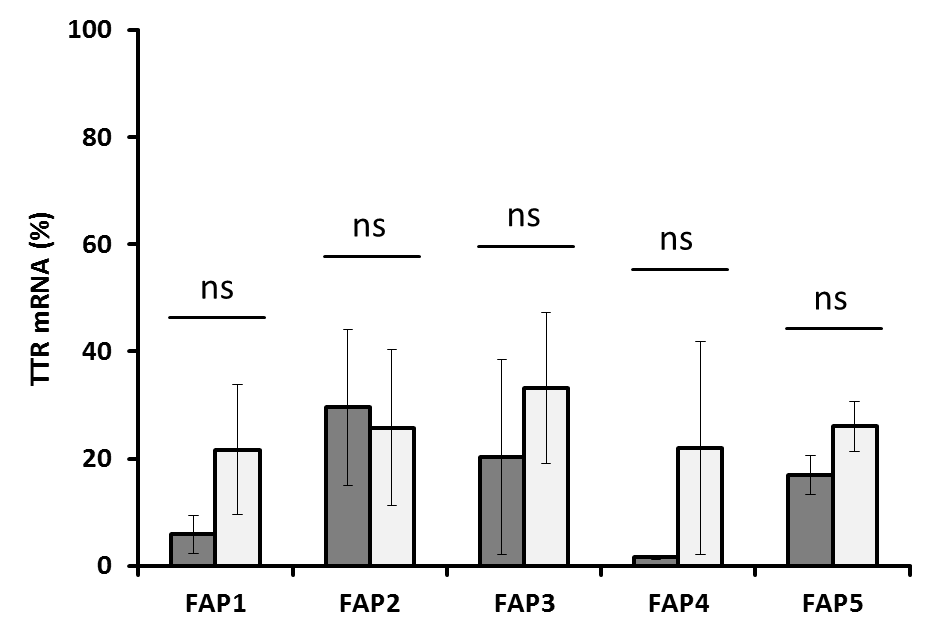
**

**S3 Fig. Clonal analysis of *TTR* knockdown in different FAP cell lines.**

Supplement: S3 Fig — TTR knockdown was assessed after treatment (24 h) with siTTR1 (grey) and TTR-ASO (white). HLCs from two iPS cell clones per FAP cell line were analyzed. Data were normalized to GAPDH. Untreated cells were set to 100%. Standard deviations are given. (DOCX) [file pone.0161455.s003.docx]

**
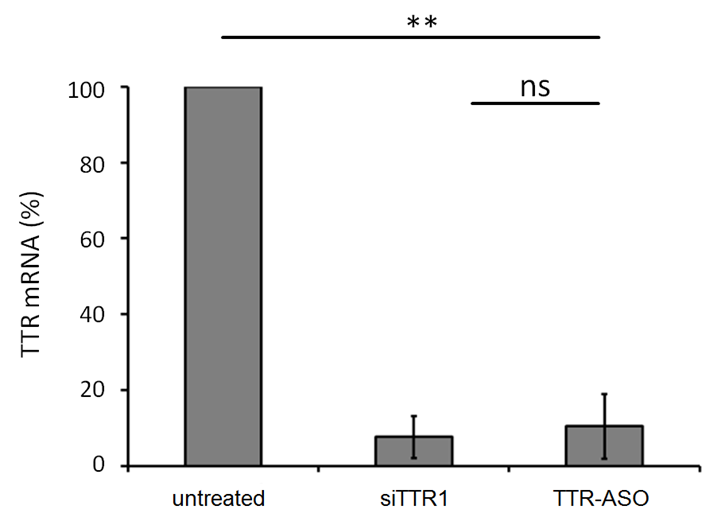
**

**S4 Fig. qRT-PCR analysis of TTR mRNA knockdown in HLCs of healthy individuals**.

Supplement: S4 Fig — Untreated cells were set to 100% (n = 3). (DOCX) [file pone.0161455.s004.docx]

**
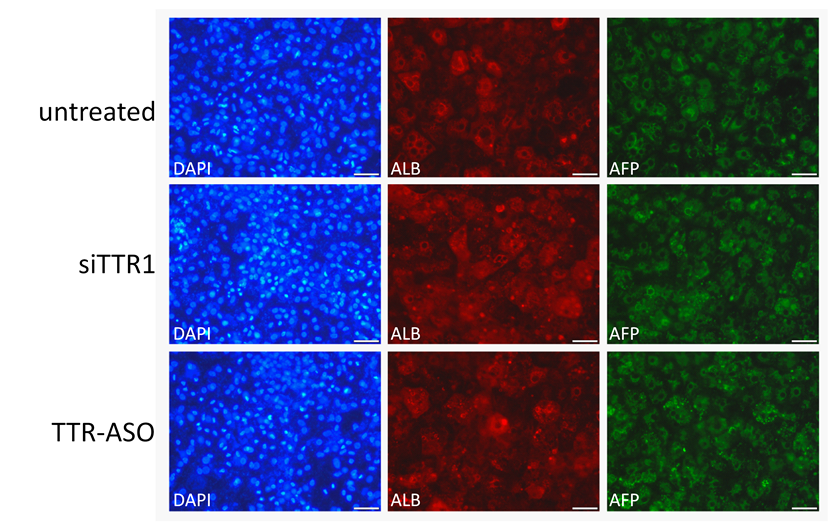
**

**S5 Fig. Protein expression of marker genes following *TTR* knockdown.**

Supplement: S5 Fig — Immunocytochemistry stainings of albumin and alpha-fetoprotein after treatment with compounds (24 h). One typical experiment derived from HLCs of FAP4 cell line is shown. Exposure time was adjusted to 1/3 second. Scale bars, 50 μM. (DOCX) [file pone.0161455.s005.docx]
