# Supplementary material for: Evaluation of Therapeutic Oligonucleotides for Familial Amyloid Polyneuropathy in Patient-Derived Hepatocyte-Like Cells
Source: PLoS One. 2016 Sep 1;11(9):e0161455. doi: 10.1371/journal.pone.0161455 (PMC5008816; doi:10.1371/journal.pone.0161455)
Supplement: S1 Table — (DOCX) [file pone.0161455.s006.docx]

**S1 Table. Antibodies used in the study.**

| **Antibody** | **Company** | **Catalog Number** |
| --- | --- | --- |
| OCT4 | Stemcell Technologies | 60093PE |
| SSEA-4 | Stemcell Technologies | 60062PE |
| TRA-1-60 | Stemcell Technologies | 60064AD |
| Nanog | Santa Cruz Biotechnology | sc-33759 |
| TTR (ICC) | Abcam | ab75815 |
| TTR (WB) | Abcam | ab16006 |
| ALB | Abcam | ab2406 |
| HNF4a | Santa Cruz Biotechnology | sc-6556 |
| AFP | Santa Cruz Biotechnology | sc-8399 |
| Alexa Fluor 594 | Life Technologies | A11012 |
| Alexa Fluor 568 | Life Technologies | A11057 |
| Alexa Fluor 488 | Life Technologies | A11001 |
| Anti IgG | GE Healthcare Life Sciences | NA934V |
