# Supplementary material for: Evaluation of Therapeutic Oligonucleotides for Familial Amyloid Polyneuropathy in Patient-Derived Hepatocyte-Like Cells
Source: PLoS One. 2016 Sep 1;11(9):e0161455. doi: 10.1371/journal.pone.0161455 (PMC5008816; doi:10.1371/journal.pone.0161455)
Supplement: S2 Table — (DOCX) [file pone.0161455.s007.docx]

**S2 Table. Primer used in the study.**

| **Name** | **Gene** | **Sequence (left/right)** | **Acc. No.** |
| --- | --- | --- | --- |
| *AFP* | Alpha-fetoprotein | TGCAATTGAGAAACCCACTG/ CTCATGGCAAAGTTCTTCCAG | NM_001134 |
| *ALB* | Albumin | GGAGATCTGCTTGAATGTGCT/ CAATGCAGTGGGATTTTTCC | NM_000477 |
| *APOA1* | Apolipoprotein A-I | GACCTTGGCCGTGCTCTTC/ GGGTTCATCTTGCTGCCAGAA | NM_000039 |
| *ASGR1* | Asialoglycoprotein receptor 1 | AGGCAATGTGGGAAGAAAGA/ AGCAGGCTGGAGTGATCTTC | NM_001197216 |
| *ATOX1* | Antioxidant 1 copper chaperone | CTGTGGAGGCTGTGCTGAAG/  TCTTGTTGGGCAGGTCAATG | NM_004045 |
| *ATP7B* | ATPase, Cu++ transporting, beta polypeptide | TCCTCTGTGTCTGTGGTGCTC/ GTATGAGGCACAGGCGCAT | NM_000053 |
| *CLDN1* | Claudin 1 | CCCAGTCAATGCCAGGTACG/  AAGTAGGGCACCTCCCAGAAG | NM_021101 |
| *SLC31A1* | Solute carrier family 31 member 1 (*CTR1*) | GTCCCAGGACCAAATGGAAC/  ACCACCTGGATGATGTGCAG | NM_001859 |
| *CYP3A5* | Cytochrome P450 family 3 subfamily A member 5 | AAGGTCAACTCCCTGTGCTG/ GGGCCTAAAGACCTTCGATT | NM_000777 |
| *CYP3A7* | Cytochrome P450 family 3 subfamily A member 7 | TATGGAACCCGTACACATGG/ AACGTCCAATAGCCCTTACG | NM_000765 |
| *FBXW2* | F-box and WD repeat domain containing 2 | TCTTGCACAGTCCTGGAGAC/ GGCTGCAGGCAGATACTTCT | NM_012164 |
| *FGA* | Fibrinogen alpha chain | AGCCCCACCCTTAGAAAAGA/  CCACTATCTGCAGTCCATGC | NM_000508 |
| *FN1* | Fibronectin 1 | CTGGCCAGTCCTACAACCAG/  TCGGGAATCTTCTCTGTCAGC | NM_001306129 |
| *FOXA2* | Forkhead box A2 | CGACTGGAGCAGCTACTATG/ GTACGTGTTCATGCCGTTCA | NM_021784 |
| *GAPDH* | Glyceraldehyde-3-phosphate dehydrogenase | CCCACTCCTCCACCTTTGAC/ CTACAGCAACAGGGTGGTGG | NM_001256799 |
| *GATA4* | GATA binding protein 4 | TCCAAACCAGAAAACGGAAG/ GAAGGCTCTCACTGCCTGAA | NM_001308093 |
| *GLUL* | Glutamate-ammonia ligase | GCTGCCATACCAACTTCAGC/  CGCTTGCTTAGTTTCTCAATGG | NM_001033044 |
| *GSTA1* | Glutathione S-transferase alpha 1 | CCGTATATTTGAGCCCAAGTGC/  TCTTCTCCAAGCCCTCAAAGC | NM_000561 |
| *HNF4A* | Hepatocyte nuclear factor 4 alpha | CAGGCTCAAGAAATGCTTCC/ GGCTGCTGTCCTCATAGCTT | NM_000457 |
| *HSPA5* | Heat shock protein family A (Hsp70) member 5 | CTGGTGTGCTCTCTGGTGATC/  TCATGACACCTCCCACAGTTTC | NM_005347 |
| *KRT7* | Keratin 7 | TGGGAGCCGTGAATATCTCTG/  GCACTGCTGGAGAAGCTCAG | NM_005556 |
| *KRT8* | Keratin 8 | GGCTCCAGGCTGAGATTGAG/  GCATCCTTAATGGCCAGCTC | NM_002273 |
| *L1CAM* | L1 cell adhesion molecule | AGGGCGGCAAATACTCAGTG/  CGCCGAAGGTCTCATCTTTC | NM_000425 |
| *NANOG* | Nanog homeobox | CAACTGGCCGAAGAATAGCAA/ CTGGGGTAGGTAGGTGCTGA | [NM_024865](http://www.ncbi.nlm.nih.gov/entrez/viewer.fcgi?db=nucleotide&val=13376297) |
| *NR1I3* | Nuclear receptor subfamily 1 group I member 3 (*CAR*) | GGTCACACACTTCGCAGACA/ GTCTTCAATGGGCAGGGAAC | NM_001077469 |
| *NR3C2* | Nuclear receptor subfamily 3 group C member 2 | ACCAACTGACCAAGCTGCTG/  CATGGGACTCTCGGAAGGTG | NM_000901 |
| *OCLN* | Occludin | GAAGCAAGTGAAGGGATCTGC/  CCAACCATCTTCTTGATGTGTG | NM_001205254 |
| *POU5F1* | POU class 5 homeobox 1 (*OCT4*) | GTGCCTGCCCTTCTAGGAAT/ CAAAAACCCTGGCACAAACT | NM_002701 |
| *SERPINA1* | Serpin peptidase inhibitor, clade A member 1 (*AAT*) | CATCACCAAGTTCCTGGAAAA/ CCCCATTGCTGAAGACCTTA | NM_000295 |
| *SLC2A1* | Solute carrier family 2 member 1 | GGGTTGTGCCATACTCATGACC/  TGGCCACGATGCTCAGATAG | NM_006516 |
| *SNAI2* | Snail family zinc finger 2 | ACCCTGGTTGCTTCAAGGAC/  GAATGGGTCTGCAGATGAGC | NM_003068 |
| *SOX17* | SRY (sex determining region Y)-box 17 | CTGCCACTTGAACAGTTTGG/ GAGGAAGCTGTTTTGGGACA | NM_022454 |
| *SOX2* | SRY (sex determining region Y)-box 2 | GCCCTGCAGTACAACTCCAT/ CTGCGAGTAGGACATGCTGTA | NM_003106 |
| *SULT2A1* | Sulfotransferase family 2A member 1 | GATGCCCATGAGAGAGGAGA/ AACGTCTTTCCCAGGAATTG | NM_003167 |
| *TF* | Transferrin | CCAGACTGTCCCACAGAACA/ GTACCATCAAGGCACAGCAA | NM_001063 |
| *TTR* | Transthyretin | GAAAGGCTGCTGATGACACC/ TCAGTTGTGAGCCCATGCAG | NM_000371 |
| *UBQLN1* | Ubiquilin 1 | GCCTCATCCCAGGGTTTACT/ GGTGTGGCGTTAGATCCATT | NM_013438 |
| *UGT1A1* | UDP glucuronosyltransferase family 1 member A1 | TGCTCATTGCCTTTTCACAG/  AAACAGCCAGACAGATGCAG | NM_000463 |
| *VIM* | Vimentin | TGGATTCACTCCCTCTGGTTG/  TCGTGATGCTGAGAAGTTTCG | NM_003380 |
